# Novel Genetic Resources in the Genus *Vigna* Unveiled from Gene Bank Accessions

**DOI:** 10.1371/journal.pone.0147568

**Published:** 2016-01-22

**Authors:** Yu Takahashi, Prakit Somta, Chiaki Muto, Kohtaro Iseki, Ken Naito, Muthaiyan Pandiyan, Senthil Natesan, Norihiko Tomooka

**Affiliations:** 1 Genetic Resources Center, National Institute of Agrobiological Sciences, Tsukuba, Ibaraki, Japan; 2 Department of Agronomy, Faculty of Agriculture at Kamphaeng Saen, Kasetsart University, Kamphaeng Saen, Nakhon Pathom, Thailand; 3 Tamil Nadu Agricultural University, Tamil Nadu, India; National University of Ireland - Galway, IRELAND

## Abstract

The genus *Vigna* (Fabaceae) consists of five subgenera, and includes more than 100 wild species. In *Vigna*, 10 crops have been domesticated from three subgenera, *Vigna*, *Plectrotropis*, and *Ceratotropis*. The habitats of wild *Vigna* species are so diverse that their genomes could harbor various genes responsible for environmental stress adaptation, which could lead to innovations in agriculture. Since some of the gene bank *Vigna* accessions were unidentified and they seemed to be novel genetic resources, these accessions were identified based on morphological traits. The phylogenetic positions were estimated based on the DNA sequences of nuclear rDNA-ITS and chloroplast *atpB-rbcL* spacer regions. Based on the results, the potential usefulness of the recently described species *V*. *indica* and *V*. *sahyadriana*, and some wild *Vigna* species, i.e., *V*. *aconitifolia*, *V*. *dalzelliana*, *V*. *khandalensis*, *V*. *marina* var. *oblonga*, and *V*. *vexillata*, was discussed.

## Introduction

The genus *Vigna*, in the family Fabaceae, comprises more than 100 wild species [1]. It is an agriculturally important taxon, which includes 10 domesticated species (crops) such as cowpea (*Vigna unguiculata* (L.) Walpers), mung bean (*Vigna radiata* (L.) Wilczek) and azuki bean (*Vigna angularis* (Willd.) Ohwi & Ohashi). Since some of their wild relatives inhabit extreme environments such as arid land, sandy beaches, and limestone karsts [2], they are expected to harbor adaptive genes, which could be used for developing stress-resistant crops for agriculturally unsuitable lands. Moreover, since they have evolved a symbiotic relationship with root-nodulating bacteria, which is also adapted to these extreme environments and contributes toward nitrogen fixation, these legumes have a high potential to contribute toward low-input sustainable agriculture [3, 4].

To introduce useful traits of wild relatives to related crops, interspecific hybridization is the most efficient and reliable strategy. Sequence-based phylogenetic relationships among species play a fundamental role as indicators to predict interspecific cross-compatibility. To increase the genetic diversity of a wild *Vigna* collection for environmental stress screening, *Vigna* accessions were introduced from several gene banks. Since some of the gene bank accessions were unidentified and seemed to be novel genetic resources that have not been analyzed at the molecular level, these accessions were identified based on morphological traits, and were included in the phylogenetic analysis.

Although Maréchal et al. [5] described seven subgenera in the genus *Vigna*, two of them, *Macrorhynchus* and *Sigmoidotropis*, have been proposed to be distinct genera, i.e., *Wajira* and *Sigmoidotropis*, respectively, based on morphological and molecular phylogenetic analyses [6, 7]. Among the five subgenera presently recognized (*Ceratotropis*, *Haydonia*, *Lasiospron*, *Plectrotropis*, and *Vigna*), crop species have been developed only from three subgenera (*Ceratotropis*, *Plectrotropis*, and *Vigna*). Therefore, we have focused on the species belonging to these subgenera in the present study.

The subgenus *Ceratotropis*, also known as the Asian *Vigna*, is agronomically the most important taxonomic group, from which seven crops have been domesticated, i.e., moth bean (*Vigna aconitifolia* (Jacq.) Maréchal), minni payaru (*Vigna stipulacea* Kuntze), mung bean, black gram (*Vigna mungo* (L.) Hepper), creole bean (*Vigna reflexo-pilosa* Hayata), rice bean (*Vigna umbellata* (Thunb.) Ohwi & Ohashi), and adzuki bean (*Vigna angularis* (Willd.) Ohwi & Ohashi). Tomooka et al. [8] described 21 species, which were divided into three sections: five species in section *Aconitifoliae* N. Tomooka & Maxted, 12 species in section *Angulares* N. Tomooka & Maxted, and four species in section *Ceratotropis* N. Tomooka & Maxted. Although four new species were recently described in the subgenus *Ceratotropis* [9–12], their molecular phylogenetic positions have not been studied. In the present study, two newly described species (*V*. *indica* and *V*. *sahyadriana*) and four wild species (wild *V*. *aconitifolia* (Jacq.) Maréchal, *Vigna dalzelliana* (O. Kuntze) Verdcourt, *Vigna khandalensis* (Santapau) Raghavan & Wadhwa, *V*. *subramaniana* (Babu ex Raizada) Raizada) of the subgenus *Ceratotropis*, which had not been used in previous molecular phylogenetic studies, were analyzed.

Maréchal et al. [5] described seven species, consisting of two sections in the subgenus *Plectrotropis* (four species in section *Plectrotropis* and three species in section *Pseudoliebrechtsia*). The subgenus *Plectrotropis* contains a lesser known but potentially important food legume called ‘tuber cowpea’ (*Vigna vexillata* (L.) A. Rich.) [13]. This fully domesticated form is still cultivated in Bali and Timor, Indonesia. Maréchal et al. [5] recognized six botanical varieties (var. *vexillata*, *angustifolia*, *doichonema*, *macrosperma*, *pluriflora*, and *yunnanensis*) in *V*. *vexillata*. Among these varieties, var. *macrosperma* was reported as a domesticated taxa but its origin was unknown. Later, considering some proposals for new treatments [14, 15], Maxted et al. [16] accepted seven taxonomic varieties in *V*. *vexillata* (var. *vexillata*, *angustifolia*, *davyi*, *dolichonema*, *lobatifolia*, *macrosperma*, and *ovata*). *V*. *vexillata* var. *davyi* and *V*. *vexillata* var. *lobatifolia* were treated as distinct species (*Vigna davyi* H. Bol., *Vigna lobatifolia* Baker) in the subgenus *Plectrotropis* in Maréchal et al. [5] *V*. *vexillata* var. *ovata* was formerly treated as *Strophostyles capensis* (Thunb.) E. Mey. As such, the taxonomic treatments of the species in the subgenus *Plectrotropis* are still considered immature, and phylogenetic discussions based on molecular sequence information are necessary. In the present study, accessions of *V*. *vexillata* var. *vexillata*, var. *angustifolia*, var. *lobatifolia*, var. *macrosperma*, var. *ovata*, and Bali domesticated accessions were analyzed.

In the subgenus *Vigna*, from which cowpea (*Vigna unguiculata*) and bambara groundnut (*Vigna subterranea*) have been domesticated, Maréchal et al. [5] described 36 species in six sections (two species in section *Catiang*, two in *Comosae*, one in *Liebrechtsia*, two in *Macrodontae*, nine in *Reticulatae*, and 20 in *Vigna*). Cowpea is classified under *Catiang*, and bambara groundnut is in the section *Vigna*. For *Vigna*, we are currently focusing on *Vigna marina* (Burm.) Merrill, which inhabits sandy beaches, as a genetic resource for salinity tolerance, and *Vigna luteola* (Jacq.) Bentham, which inhabits riversides, as a flood-tolerant genetic resource [17, 18]. These two species are closely related, and Padulosi and Ng [19] described *V*. *marina* ssp. *oblonga* Padulosi as being distributed in coastal areas of West Africa. Sonnante et al. [20] confirmed the genetic independence of *V*. *luteola*, *V*. *marina* ssp. *marina*, and *V*. *marina* ssp. *oblonga* based on isozymes and RAPD. In addition, they showed that *V*. *marina* ssp. *oblonga* was more closely related to *V*. *luteola* than to *V*. *marina* ssp. *marina*. However, *V*. *marina* ssp. *oblonga* was not included in subsequent phylogenetic analysis based on DNA sequences, although Pasquet et al. [15] described *V*. *marina* ssp. *oblonga* as being a synonym of *V*. *luteola*.

We therefore performed a phylogenetic characterization of the aforementioned taxa. To our knowledge, a phylogenetic study using DNA sequences had not been conducted on these taxa based on the DNA sequences of the internal transcribed spacer region of the ribosomal DNA on the nuclear genome (hereafter rDNA-ITS), and the *atpB-rbcL* intergenic spacer on the chloroplast genome (hereafter *atpB-rbcL*).

## Materials and Methods

### Plant materials

Seventy-one accessions of the genus *Vigna*, consisting of 28 species and three subgenera (*Ceratotropis*, *Plectrotropis*, and *Vigna*) conserved at the National Institute of Agrobiological Sciences, Japan, were used (Table 1). Originally, nine accessions were either unidentified, or seemed to be misidentified, as shown by the bold texts in Table 1. For the morphological analysis and DNA extraction, all the accessions were planted in six 0.3-L plastic pots (one seed/pot), and a 5-L plastic pot (six seeds/pot), and kept in a greenhouse where the temperature was maintained above 20°C with 12 hours of day length. The morphology of each plant was evaluated. For *V*. *aconitifolia*, weight of a hundred grains, pod shattering, and water absorbency of the seed were evaluated as domesticated traits. When evaluating pod shattering, 20 pods were dried overnight in a circulating incubator at 40°C. Twenty seeds were submerged in a Petri dish at 25°C for two days, and the number of seeds that absorbed water was recorded. We used common bean (*Phaseolus vulgaris* cv. Taisho-kintoki) as an outgroup for molecular phylogenetic analysis.

**Table 1:** Plant materials used for phylogenetic analysis, and the sequence length and accession No. of rDNA-ITS and *atpB-rbcL*. deposited at DDBJ.

ID	Section	Species Name	Status	Origin	JP No.	Original Conservation Site	Original ID and Species Identification	rDNA-ITS Sequence Length (bp)	rDNA-ITS DDBJ Accession No.	*atpB-rbcL Sequence Length (bp)*	*atpB-rbcL DDBJ Accession No*.
	Subgenus Ceratotropis										
1	Aconitifoliae	V. aconitifolia	Domesticated	India	245857	TNAU GB	2009TN58	562	LC082015	*700*	*LC082267*
2	Aconitifoliae	V. aconitifolia	Domesticated	India	245897	TNAU GB	2009TN99	562	LC082017	*699*	*LC082269*
3	Aconitifoliae	V. aconitifolia	Domesticated	Pakistan	104332	NIAS GB	2752(5)	562	LC082016	*699*	*LC082268*
4	Aconitifoliae	V. aconitifolia	Wild	India	235416	Australian GB	AUSTRCF106324, Vigna sp.	562	LC082014	*699*	*LC082266*
5	Aconitifoliae	V. aconitifolia	Wild	India	245864	TNAU GB	2009TN66, Vigna sp.	562	LC082012	*699*	*LC082264*
6	Aconitifoliae	V. aconitifolia	Wild	India	245865	TNAU GB	2009TN67, Vigna sp.	562	LC082013	*700*	*LC082265*
7	Aconitifoliae	V. aridicola	Wild	Sri Lanka	205894	NIAS GB	2000S-11	561	LC082018	*689*	*LC082270*
8	Aconitifoliae	V. aridicola	Wild	Sri Lanka	205896	NIAS GB	2000S-2	561	LC082019	*689*	*LC082271*
9	Aconitifoliae	V. aridicola	Wild	Sri Lanka	207977	NIAS GB	2001SL-28	561	LC082020	*690*	*LC082272*
10	Aconitifoliae	V. indica	Wild	India	235417	ILRI GB	IL-25019, V. trilobata	562	LC082011	*697*	*LC082263*
11	Aconitifoliae	V. khandalensis	Wild	India	253828	TNAU GB	VC76	561	LC082005	*687*	*LC082257*
12	Aconitifoliae	V. stipulacea	Domesticated	India	245503	TNAU GB	2008TN29	561	LC082007	*690*	*LC082259*
13	Aconitifoliae	V. stipulacea	Wild	Sri Lanka	205892	NIAS GB	2000S-6	562	LC082006	*690*	*LC082258*
14	Aconitifoliae	V. subramaniana	Wild	India	229278	Australian GB	AUSTRCF106193, Vigna sp.	562	LC064351	*696*	*LC064361*
15	Aconitifoliae	V. subramaniana	Wild	India	229284	Australian GB	AUSTRCF85155, V. radiata var. sublobata	562	LC064350	*697*	*LC064360*
16	Aconitifoliae	V. trilobata	Wild	India	245881	TNAU GB	2009TN83	562	LC082010	*690*	*LC082262*
17	Aconitifoliae	V. trilobata	Wild	Sri Lanka	210605	NIAS GB	2000S-5-1	562	LC082009	*690*	*LC082261*
18	Aconitifoliae	V. trilobata	Wild	Sri Lanka	205895	NIAS GB	2000S-13	562	LC082008	*690*	*LC082260*
19	Angulares	V. angularis var. angularis	Domesticated	Japan	37752	NIAS GB	ERIMOSHOUZU	557	LC081992	*688*	*LC082244*
20	Angulares	V. angularis var. nipponensis	Wild	Japan	87910	NIAS GB	CED96101602	557	LC081993	*688*	*LC082245*
21	Angulares	V. angularis var. nipponensis	Wild	Laos	226665	NIAS GB	2005L34	557	LC081995	*688*	*LC082247*
22	Angulares	V. dalzelliana	Wild	India	235419	Australian GB	AUSTRCF85146	557	LC081997	*689*	*LC082249*
23	Angulares	V. dalzelliana	Wild	Myanmar	210811	NIAS GB	2001M24, Vigna sp.	557	LC081996	*696*	*LC082248*
24	Angulares	V. exilis	Wild	Thailand	205884	NIAS GB	99T-10-1	557	LC081985	*690*	*LC082237*
25	Angulares	V. hirtella	Wild	Sri Lanka	218935	NIAS GB	9902–48	557	LC081984	*690*	*LC082236*
26	Angulares	V. hirtella	Wild	Thailand	109681	NIAS GB	CED891122-(9)	557	LC081983	*691*	*LC082235*
27	Angulares	V. hirtella	Wild	Laos	220137	NIAS GB	2003L-14	558	LC081988	*689*	*LC082240*
28	Angulares	V. hirtella	Wild	Thailand	108562	NIAS GB	96120305	563	LC081989	*689*	*LC082241*
29	Angulares	V. minima	Wild	Thailand	107869	NIAS GB	CED891125-(10)	556	LC081998	*690*	*LC082250*
30	Angulares	V. minima	Wild	Indonesia	218938	Belgian GB	NI1363	556	LC082000	*690*	*LC082252*
31	Angulares	V. minima	Wild	Papua N.G.	226877	NIAS GB	2005PNG15	556	LC081999	*692*	*LC082251*
32	Angulares	V. nakashimae	Wild	Japan	107879	NIAS GB	Ukushima	556	LC082002	*693*	*LC082254*
33	Angulares	V. nepalensis	Wild	Nepal	107881	NIAS GB	Nepalen	557	LC081994	*689*	*LC082246*
34	Angulares	V. reflexo-pilosa var. glabra	Domesticated	Philippines	109684	AVRDC GB	V1160	557	LC081986	*698*	*LC082238*
35	Angulares	V. reflexo-pilosa var. reflexo-pilosa	Wild	Malaysia	108867	NIAS GB	510–1	557	LC081987	*698*	*LC082239*
36	Angulares	V. riukiuensis	Wild	Japan	108810	NIAS GB	Y-4-1	556	LC082001	*692*	*LC082253*
37	Angulares	V. tenuicaulis	Wild	Myanmar	227438	NIAS GB	KYONKADON	557	LC081991	*688*	*LC082243*
38	Angulares	V. tenuicaulis	Wild	Thailand	109682	NIAS GB	CED891122-(8)	557	LC081990	*688*	*LC082242*
39	Angulares	V. trinervia	Wild	Malaysia	108840	NIAS GB	503–4	561	LC064352	*698*	*LC064362*
40	Angulares	V. umbellata	Domesticated	Japan	99485	NIAS GB	Menaga	557	LC081982	*689*	*LC082234*
41	Angulares	V. umbellata	Wild	Thailand	210639	NIAS GB	99T-2	557	LC064307	*689*	*LC064328*
42	Angulares	V. umbellata	Wild	Thailand	109675	NIAS GB	(6)-1-1	557	LC081981	*689*	*LC082233*
43	Angulares	Vigna sp.	Wild	Thailand	210644	NIAS GB	99T-9	557	LC064303	*689*	*LC064324*
44	Ceratotropis	V. grandiflora	Wild	Thailand	107862	NIAS GB	CED891119-(1)	562	LC064345	*694*	*LC064355*
45	Ceratotropis	V. mungo var. mungo	Domesticated	Thailand	109668	NIAS GB	Subsomotod	562	LC064346	*689*	*LC064356*
46	Ceratotropis	V. mungo var. silvestris	Wild	India	107874	NBPGR	TC2211	562	LC064347	*690*	*LC064357*
47	Ceratotropis	V. radiata var. radiata	Domesticated	Thailand	110830	NIAS GB	CN60	595	LC064348	*688*	*LC064358*
48	Ceratotropis	V. radiata var. sublobata	Wild	Madagascar	107877	AVRDC GB	TC1966	587	LC064349	*688*	*LC064359*
49	Ceratotropis	V. radiata var. sublobata	Wild	Papua N.G.	226874	NIAS GB	2005PNG08	597	LC082004	*688*	*LC082256*
50	Ceratotropis	V. sahyadriana	Wild	India	235420	Australian GB	AusTRCF104896, Vigna sp.	568	LC082003	*689*	*LC082255*
51	Ceratotropis	Vigna sp.	Wild	India	110836	Belgian GB	NI 1135, V. radiata var. setulosa	564	LC064353	*688*	*LC064363*
52	Ceratotropis	Vigna sp.	Wild	India	245506	TNAU GB	2008TN32, V. hainiana	559	LC064354	*688*	*LC064364*
	Subgenus Plectrotropis										
53	Plectrotropis	V. vexillata	Domesticated	Indonesia	235863	Belgian GB	NI 1858	560	LC082032	*683*	*LC082284*
54	Plectrotropis	V. vexillata	Wild	Brazil	202337	USDA GB	PI 406391	562	LC082035	*684*	*LC082287*
55	Plectrotropis	V. vexillata	Wild	Papua N.G.	230747	NIAS GB	2006PNG-37	563	LC082037	*683*	*LC082289*
56	Plectrotropis	V. vexillata	Wild	Suriname	202334	USDA GB	PI 406383	563	LC082036	*684*	*LC082288*
57	Plectrotropis	V. vexillata var. angustifolia	Wild	Columbia	235869	Belgian GB	NI 936	563	LC082038	*684*	*LC082290*
58	Plectrotropis	V. vexillata var. lobatifolia	Wild	Namibia	235903	Belgian GB	NI 546	557	LC082031	*686*	*LC082283*
59	Plectrotropis	V. vexillata var. macrosperma	Domesticated	Sudan	235905	Belgian GB	NI 111	559	LC082034	*684*	*LC082286*
60	Plectrotropis	V. vexillata var. ovata	Wild	South Africa	235908	Belgian GB	NI 1869	562	LC082033	*684*	*LC082285*
61	Plectrotropis	V. vexillata var. vexillata	Wild	Congo	235912	Belgian GB	NI 245	563	LC082039	*684*	*LC082291*
	Subgenus Vigna										
62	Catiang	V. unguiculata	Domesticated	Nigeria	86801	IITA GB	IT 84S 2246	581	LC082027	*686*	*LC082279*
63	Catiang	V. unguiculata	Domesticated	Sudan	86877	IITA GB	TVU 11979	581	LC082026	*686*	*LC082278*
64	Catiang	V. unguiculata	Domesticated	Sudan	86879	IITA GB	TVU 11986	581	LC082028	*686*	*LC082280*
65	Catiang	V. unguiculata ssp. dekindtiana	Wild	Mali	89083	IITA GB	TVNU 457	575	LC082030	*684*	*LC082282*
66	Catiang	V. unguiculata ssp. sesquipedalis	Domesticated	Sri Lanka	81610	NIAS GB	MA	581	LC082029	*686*	*LC082281*
67	Vigna	V. luteola	Wild	Australia	236246	Australian GB	AUSTRCF 320527	566	LC082021	*689*	*LC082273*
68	Vigna	V. luteola	Wild	Brazil	235855	Belgian GB	NI 858	566	LC082023	*689*	*LC082275*
69	Vigna	V. marina ssp. marina	Wild	Japan	235813	NIAS GB	2009IRIO-1	569	LC082022	*690*	*LC082274*
70	Vigna	V. marina ssp. oblonga	Wild	Benin	233389	NIAS GB	2006BENIN29	567	LC082024	*690*	*LC082276*
71	Vigna	V. subterranea	Domesticated	unknown	79992	NIAS GB	L15-20-2	575	LC082025	*690*	*LC082277*
72	-	Phaseolus vulgaris	Domesticated	Japan	219310	NIAS GB	TAISHOU KINTOKI	554	LC082303	*679*	*LC082302*

Nine accessions which were originally either unidentified, or seemed to be misidentified are shown by bold texts.

### DNA Sequencing

We sequenced the rDNA-ITS and *atpB-rbcL* of 72 accessions. DNA was extracted from young leaves using a modified CTAB method [21]. PCR primers were designed according to the previous study [22]; C2 (5’-TCCTCCGCTTATTGATATGC-3’) and G1 (5’-GGAAGGAGAAGTCGTAACAAGG-3’) for rDNA-ITS, and AT1 (5’-AGAACCAGAAGTAGTAGGAT-3’) and RB (5’-ACACCAGCTTTGAATCCAAC-3’) for *atpB-rbcL*. The PCR mixture, containing KOD-Plus-Neo one unit (TOYOBO), 1 x PCR Buffer supplied by the manufacturer, 200 μM dNTPs, 1.5 mM MgSO_4_, 10 ng of the DNA template, and 0.2 μM of each primer pair, was prepared in a total volume of 50 μL. The PCR cycle was as follows: 94°C for 2 min, 35 cycles of 98°C for 10 sec and 68°C for 1 min. The amplified PCR product was mixed with 2 μL of ExoSAP-IT, which had been diluted 20-fold, and incubated at 37°C for 30 min, and 80°C for 15 min. The sequencing reaction was conducted according to the protocol of BigDye Terminator v3.1 Cycle Sequencing Kit (Applied Biosystems). The reactant was precipitated using ethanol, dried, and dissolved in 10 μL Hi-Di Formamid. The mixture was treated at 95°C for 5 min, and the DNA sequence was determined using a ABI PRISM 3130xl DNA Analyzer (Applied Biosystems). Sequencing was repeated until the depth of each base was greater than five, and the nucleotide sequence was determined according to majority rule in cases where a single nucleotide polymorphism was present. The accession numbers of the sequence information deposited in the DNA Data Bank of Japan (www.ddbj.nig.ac.jp/) are shown in Table 1.

Multiple alignment was conducted for each rDNA-ITS and *atpB-rbcL* using Clustal W [23]. The sequence frame was determined according to the previous study [22], and the trimmed sequence was used to construct a phylogenetic tree by the maximum likelihood estimation using MEGA6 [24]. Bootstrap analysis was conducted with 1000 replications.

## Results

### Morphology-based species identification

Among the nine unidentified or misidentified accessions, six accessions were identified as the following four species (*V*. *aconitifolia*, *V*. *dalzelliana*, *V*. *indica*, and *V*. *sahyadriana*) based on morphological observation.

**Fig 1:**
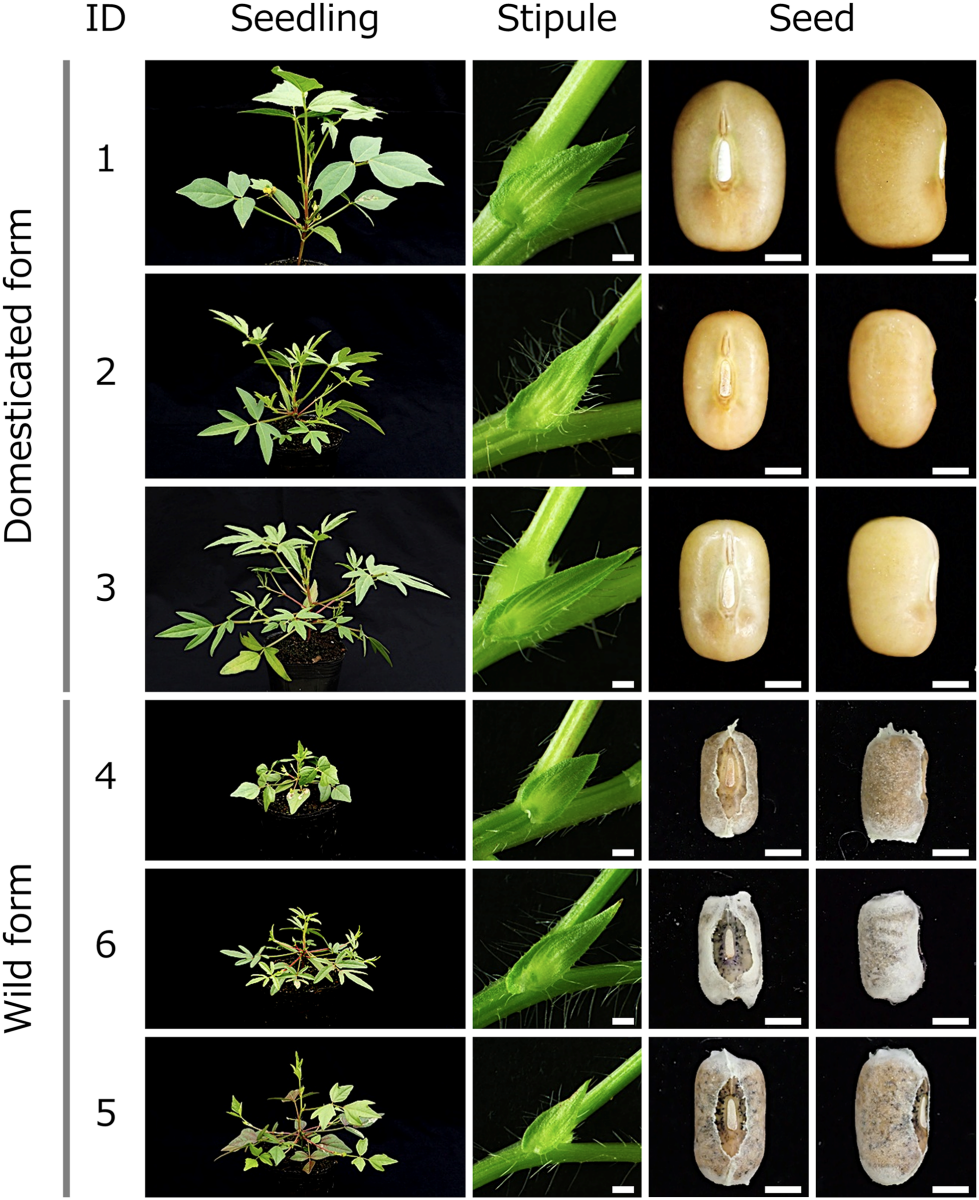
Domesticated form and wild ancestral form of moth bean (*V*. *aconitifolia*). Scale bars are 1 mm.

Accessions ID-4, ID-5, and ID-6, which were collected in India, were identified as the wild forms of moth bean (*V*. *aconitifolia*). Seedling, stipule, and seed morphologies of the domesticated and newly identified wild forms of *V*. *aconitifolia* are shown in Fig 1. Both domesticated and wild forms showed similar variations in leaflet shape, ranging from entire to deeply lobed. Only seeds of the wild forms were covered with a semi-transparent seed coat covering. While the domesticated forms were characterized by larger seeds with water-permeable seed coat and non-shattering pods, the wild forms were found to have smaller seeds, with a water-proof seed coat and high shattering pods (Table 2).

**Table 2:** Comparison of domestication related traits in domesticated and wild *V*. *aconitifolia*.

ID	Status	Seed weight ± SD (g/100 grains)^1^	Shattering pods (%)	Germination (%)
1	Domesticated	3.39 ± 0.42 a	0	100
2	Domesticated	2.03 ± 0.38 b	0	100
3	Domesticated	2.20 ± 0.11 b	0	100
4	Wild	0.86 ± 0.14 c	100	0
5	Wild	1.15 ± 0.08 c	100	0
6	Wild	1.26 ± 0.11 c	100	0

^1^ Averages of 3 replications. Different letters indicate that seed weights are significantly different, by Tukey—Kramer’s HSD test (P < 0.05).

**Fig 2:**
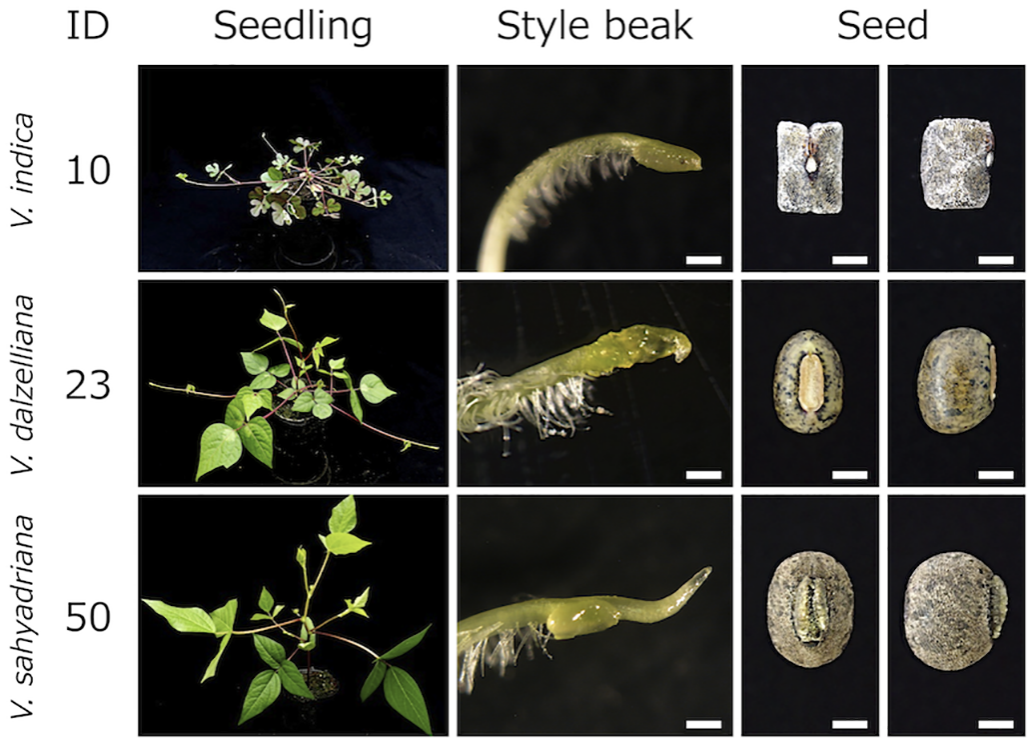
Morphological characteristics of *V*. *dalzelliana*, *V*. *sahyadriana*, and *V*. *indica*. Scale bars are 0.3 mm with style beak, and 1 mm with seeds.

Morphologies of the seedling, style beak, and seed of the remaining accessions newly identified as *V*. *dalzelliana*, *V*. *indica*, and *V*. *sahyadriana* are shown in Fig 2. Accession ID-23, collected in southern Myanmar, showed hypogeal germination with petiolate primary leaves, glabrous pods, seeds without seed coat coverings (smooth seed coat), small yellow flowers, left curved keel petal with protuberance on left keel (keel pocket), indicating that this accession belonged to the section *Angulares* in the subgenus *Ceratotropis*. Additionally, it had a flat style beak (spoon-like shape), which is a key characteristic of *V*. *dalzelliana*. Therefore, we have identified this accession as *V*. *dalzelliana*.

Accession ID-50, collected in India, was introduced from the gene bank of Australia (AusTRCF104896), where it was treated as *Vigna* sp. (Table 1). It showed epigeal germination with sessile primary leaves, seeds with seed coat covering, hairy pods, yellow flower, and left curved keel petal with a prominent protuberance on the left keel petal (keel pocket), indicating that this accession belongs to the section *Ceratotropis* in the subgenus *Ceratotropis*. Seed morphology and very long style beak matched the characteristics of *V*. *mungo*, whereas the direction of laterally attaching pods to the peduncle matched that of *V*. *radiata*. These characteristics matched the key characters of *V*. *sahyadriana* well, which was described as a new species by Aitawade et al. [10].

Accession ID-10, collected in India, was introduced from the ILRI (International Livestock Research Institute) gene bank (IL-25019), where it was conserved as *V*. *trilobata*. It showed epigeal germination with sessile primary leaves, seeds with seed coat covering, hairy pods, small yellow flowers, left curved keel petal with a small protuberance on left keel petal (keel pocket), and a protruding growth habit with deeply lobed leaflets, indicating this accession belongs to the section *Aconitifoliae* in the subgenus *Ceratotropis*. At a glance, it had a very similar overall morphology to *V*. *trilobata*. However, its stipule was lanceolate, and its seed was rectangular with a very short, non-protruding hilum, which did not match the key characters of *V*. *trilobata*. These characteristics matched those of *V*. *indica*, which was described as a new species by Dixit et al. [9].

Accession ID-43, collected in Thailand, was originally identified as *V*. *umbellata*. However, it showed some features that did not match the key characteristics of *V*. *umbellata*. Accession ID-51, collected in northern India, was introduced from a Belgian gene bank (NI 1135) as *V*. *radiata* var. *setulosa*. Accession ID-52, collected in southern India, was introduced from the Tamil Nadu Agricultural University (TN32) as *V*. *hainiana*. Both of these accessions had a similar morphology to that of *V*. *radiata* in general. However, they showed some features that did not match the key characteristics of *V*. *radiata*. Therefore, we could not determine the taxonomic identification for these three accessions based on the morphological analysis in the present study.

### Molecular phylogenetic analysis

DNA sequences of rDNA-ITS and *atpB-rbcL* were determined for 71 accessions of the genus *Vigna*. For rDNA-ITS, the total length ranged from 556–597 bp; *V*. *minima*, *V*. *riukiuensis*, and *V*. *nakashimae* had the shortest (556 bp), and *V*. *radiata* had the longest rDNA-ITS (587–597 bp). The total lengths of *atpB-rbcL* ranged from 683 to 700 bp; *V*. *unguiculata* and *V*. *vexillata* had the shortest (683–686 bp), whereas *V*. *aconitifolia* had the longest *atpB-rbcL* (699–700 bp) (Table 1). The numbers of polymorphic sites in rDNA-ITS and *atpB-rbcL* were 211 and 80, respectively.

**Fig 3:**
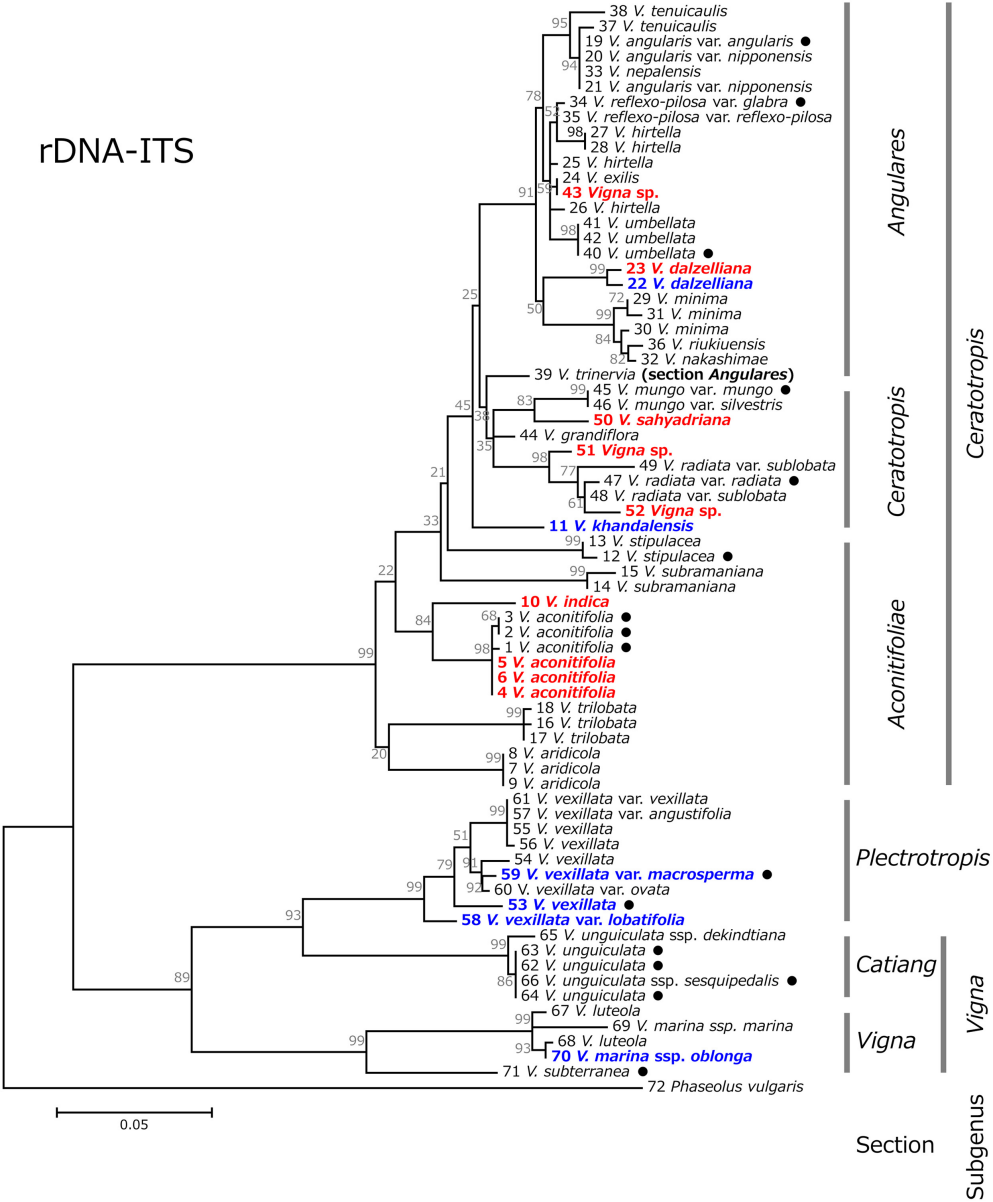
Maximum likelihood tree based on nuclear rDNA-ITS region for the genus *Vigna*, with *Phaseolus vulgaris* as an outgroup. Numbers beside branches represent bootstrap values (%) based on 1000 replications. Scale indicates genetic distance. Domesticated accessions are indicated with black circles, accessions which have been introduced as unidentified or misidentified accessions are indicated with red text, and taxa in which phylogenetic discussion using DNA sequences had not been conducted are indicated with blue text.

**Fig 4:**
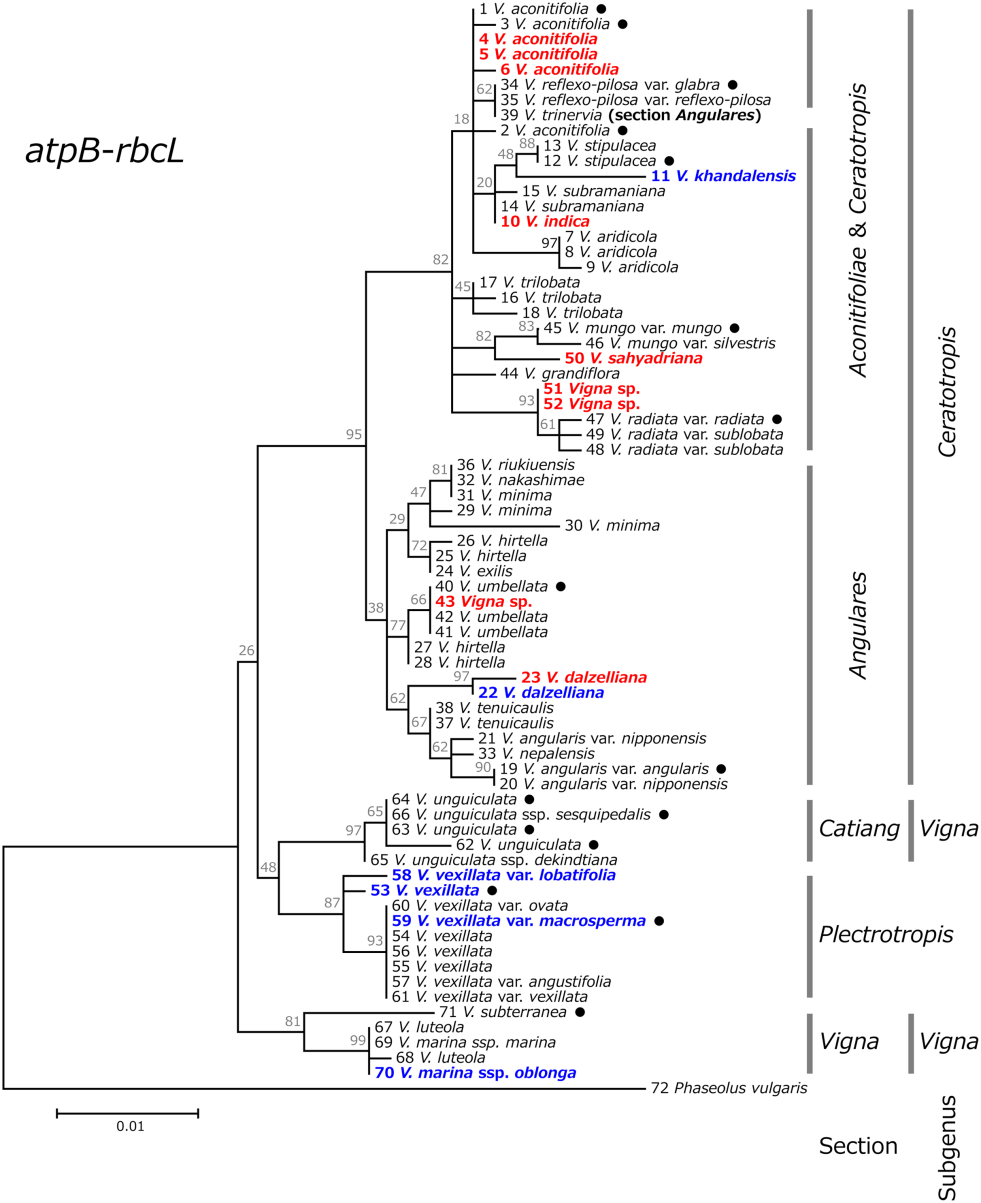
Maximum likelihood tree based on chloroplast *atpB-rbcL* spacer region for the genus *Vigna*, with *Phaseolus vulgaris* as an outgroup. Numbers beside branches represent bootstrap values (%) based on 1000 replications. Scale indicates genetic distance. Domesticated accessions are indicated with black circles, accessions which have been introduced as unidentified or misidentified accessions are indicated with red text, and taxa of which phylogenetic discussion using DNA sequences had not been conducted are indicated with blue text.

Based on these sequences of rDNA-ITS and *atpB-rbcL*, phylogenetic trees for respective regions were constructed (Figs 3 and 4). In both phylogenetic trees, the subgenus *Ceratotropis* formed a single cluster, distinct from the subgenera *Vigna* and *Plectrotropis*. The section *Catiang* in the subgenus *Vigna* allied with the subgenus *Plectrotropis* forming a single cluster, while the section *Vigna* in the subgenus *Vigna* was distantly allied.

The phylogenetic tree based on rDNA-ITS divided the section *Aconitifoliae* into multiple branches, and clustered the section *Ceratotropis* and *Angulares* independently (Fig 3). Alternatively, the phylogenetic tree based on *atpB-rbcL* divided the subgenus *Ceratotropis* into two groups, i.e., a blended group comprising the sections *Aconitifoliae* and *Ceratotropis*, and the section *Angulares* (Fig 4). While the section *Angulares* clustered distinctly from other groups, the interspecific genetic distances within the *Angulares* cluster were small.

The phylogenetic analysis revealed the species most closely related to the accessions that were newly identified in this study. Accession ID-4, ID-5, and ID-6, identified as a wild form of moth bean, were most closely related to moth bean (*V*. *aconitifolia*). Accession ID-23 (Myanmar), identified as *V*. *dalzelliana*, was most closely related to the *V*. *dalzelliana* collected in India. Accession ID-50, identified as *V*. *sahyadriana*, was most closely related to *V*. *mungo*. Accession ID-10, identified as *V*. *indica*, was most closely related to *V*. *aconitifolia* in the phylogenetic tree based on rDNA-ITS, and to *V*. *subramaniana* in the phylogenetic tree based on *atpB-rbcL*. Accession ID-43 (*Vigna* sp.) was closely related to *V*. *exilis* in the rDNA-ITS tree, whereas it was allied with *V*. *umbellata* in the *atpB-rbcL* tree. Accessions ID-51 and ID-52 were most closely related to *V*. *radiata* in both trees.

*V*. *khandalensis* (accession ID-11) was differentiated substantially from other species, but was relatively close to *V*. *stipulacea*. Accessions within *V*. *vexillata* showed considerable levels of genetic variation. The accession ID-58 (*V*. *vexillata* var. *lobatifolia*), and the Indonesian domesticated form (accession ID-53) noticeably differentiated from other *V*. *vexillata* accessions. *V*. *marina* ssp. *oblonga* (accession ID-70), which was found on the coast of West Africa, was more closely related to *V*. *luteola* than to *V*. *marina* ssp. *marina*.

## Discussion

### Genetic differentiation within the genus *Vigna*

The subgenus *Ceratotropis* is thought to have emerged from the subgenus *Vigna* via the subgenus *Plectrotropis* [16, 25, 26]. The theoretical basis of this hypothesis is that, while the subgenus *Vigna* has a symmetric keel without pocket, the subgenus *Plectrotropis* has a curved keel with a pocket, and the subgenus *Ceratotropis* has a more prominently twisted keel with a more prolonged pocket. However, the phylogenetic tree using rDNA-ITS in this study suggested the following genetic differentiation patterns. The common ancestor of the genus *Vigna* first diverged into the common ancestor of the subgenera *Vigna* plus *Plectrotropis*, and the common ancestor of the subgenus *Ceratotropis*. Then, the common ancestor of the subgenera *Vigna* plus *Plectrotropis* diverged into the common ancestor of the section *Vigna* (subgenus *Vigna*) and the common ancestor of the section *Catiang* (subgenus *Vigna*) plus subgenus *Plectrotropis*. This is supported by the fact that the species in the section *Catiang* (subgenus *Vigna*) and the subgenus *Plectrotropis* have purple flowers, while those in the section *Vigna* (subgenus *Vigna*) have yellow flowers. Similar species relationships to our phylogenetic tree were obtained in previous studies using other molecular markers [7, 20, 27]. Therefore, it seems more appropriate to raise the rank of the section *Catiang* as a subgenus level. However, we leave this taxonomic revision for future work, since we used the limited number of species in the section *Catiang*, *Vigna*, and the subgenus *Plectrotropis*.

“*Plectrotropis*”, which represents the subgenus, and the section including *V*. *vexillata*, has been misspelled as “*Plectotropis*” in Maréchal et al. [5], and in many subsequent publications such as Tomooka et al. [8] and Maxted et al. [16], but the former should be the correct spelling, as it appeared in Schumach [28] and Baker [29] as a genus name and a subgenus name, respectively.

After cowpea and *V*. *vexillata* were shown to be relatively close to each other by molecular analysis [30], an interspecific hybrid between the two species was obtained [31]. Moreover, an interspecific hybrid was obtained between cowpea and *V*. *luteola*, which are more distantly related species [32]. In the present study, we propose that *V*. *marina* is worth trying for producing interspecific hybrids with bambara groundnut (*V*. *subterranea*), based on their relatively close phylogenetic positions. *V*. *marina* is highly tolerant to salinity and alkaline soil [17, 33], while bambara groundnut is a crop that is adapted to arid lands [34]. Drought, saline, and alkaline soils are the most important environmental stresses to be addressed in agriculture.

### Novel genetic resources in the genus *Vigna*

#### *Vigna indica* T.M. Dixit, K.V. Bhat & S.R. Yadav

Accession ID-10 is revealed to be the only germplasm of *V*. *indica* currently available at the gene bank. Although a holotype (*Rothe 6229a*) of this species was described as *V*. *trilobata* (L.) Verdcourt var. *pusilla* Naik et Pokle [35], results of the phylogenetic analysis supported Dixit et al. [9], in that this taxon is an independent species in the section *Aconitifoliae*. Whereas *V*. *indica* was reported to be morphologically most similar to *V*. *aridicola* by Dixit et al. [9], it was also similar to the wild form of *V*. *aconitifolia* in its stipule and flower morphology.

In this study, *V*. *indica* showed the closest relationship with *V*. *aconitifolia* in the rDNA-ITS tree. Conversely, it showed almost the same *atpB-rbcL* sequence as that of *V*. *subramaniana*. These facts suggest the possibility that *V*. *indica* is derived from an interspecific hybrid between *V*. *subramaniana* and *V*. *aconitifolia*. Further studies are necessary to confirm the origin of this species. Additionally, useful traits screening and interspecific cross-compatibility of *V*. *indica* should be conducted to determine its usefulness as a genetic resource, especially for moth bean (*V*. *aconitifolia*), the most closely related crop.

#### *Vigna sahyadriana* Aitawade, K.V. Bhat et S.R. Yadav

Accession ID-50 is the only germplasm of *V*. *sahyadriana* available from the gene bank at present. This species was recently described as a new species distributed in Maharashtra, India [10]. Since accession ID-50 was collected in Madhya Pradesh, India, the distribution range of this species seems to have expanded toward the inland of India.

Accession ID-50 was most closely related to, but clearly distinguishable from, black gram (*V*. *mungo*) in both phylogenetic trees (Figs 3 and 4). This suggests that the useful traits and interspecific cross-compatibility of *V*. *sahyadriana* should be investigated to determine if it can be used as genetic resources for black gram.

#### *Vigna aconitifolia* (Jacq.) Maréchal: Wild ancestor of moth bean

Although the wild form of moth bean was documented to be distributed in India [36], living samples have not been identified in the gene bank [27], and therefore its identity and useful traits have not been studied. In this study, we found the wild ancestor of moth bean in a gene bank collection. Accessions ID-5 and ID-6 were collected in Tamil Nadu, and accession ID-4 was collected in Andhra Pradesh, India. The collection sites of these three accessions suggest that the primary habitat of the wild form of moth bean is southeastern India.

Moth beans have been cultivated mainly in arid lands from India to Pakistan, and also in some other counties including Bangladesh, Myanmar, and China [37]. Since moth bean is reported as a crop most tolerant to drought and heat in the subgenus *Ceratotropis* [38, 39], it is generally thought to be suitable as a crop in tropical arid lands.

Recently, we have found that the wild ancestor of moth bean showed higher drought tolerance than the domesticated forms, and we successfully obtained the F_2_ lines among the two forms (data not shown). Moreover, since the interspecific hybrid between mung bean and moth bean has been reported [40], wild moth bean would be useful to develop moth bean and mung bean varieties with higher drought tolerance.

#### *Vigna dalzelliana* (O. Kuntze) Verdcourt

The geographical distribution of this species was thought to be limited to India and Sri Lanka [8]. Although Thuan [41] reported *V*. *dalzelliata* in the Indo-China region (Vietnam, Laos, and Cambodia), it was the result of a misidentification of *V*. *minima* specimens [39]. More recently, John et al. [42] reported that they found *V*. *dalzelliana* in the Andaman Islands. Identification of accession ID-23 as *V*. *dalzelliana* in this study revealed an additional range of geographical distribution for this species, southern Myanmar.

The dissemination pathway of *V*. *dalzelliana* from India to southern Myanmar is unknown. Further explorations in the broad areas along the Bengal Gulf (Bangladesh and Myanmar) are necessary. However, since *V*. *dalzelliana* also inhabits Sri Lanka and the Andaman Islands [8, 42], researchers must consider the possibility that the distribution range expanded from India to Myanmar via these Islands.

Based on the rDNA-ITS tree, *V*. *dalzelliana* is located at the basal position with a *V*. *minima* species complex (*V*. *minima*, *V*. *nakashimae*, *V*. *riukiuensis*) [43], and both of these species are well differentiated within the section *Angulares* (Fig 3). Since *V*. *dalzelliana* is the only species known to be distributed in south India, where species of the other two sections are rich, it could be the ancestral species of the section *Angulares*. Investigating the process of the species emergence and expansion will provide important insights to understand the evolution of this section.

#### *Vigna khandalensis* (Santapau) Raghavan & Wadhwa

*Vigna khandalensis* was reported to inhabit a rainforest climate area in the Western Ghats and the Deccan Plateau in India [44]. It is the only wild species to have an erect plant type in the subgenus *Ceratotropis* in *Vigna*. Its seeds were collected as a food during famines [45]. While Tomooka et al. [8] classified this species in the section *Aconitifoliae* based on the short keel pocket and style beak; Bisht et al. [46] reported that this species is morphologically similar to species in the section *Ceratotoropis*. The phylogenetic trees in this study suggested that *V*. *khandalensis* is a species in the section *Aconitifolia*, and located at the basal position to the species in the section *Ceratotoropis*. *V*. *khandalensis* was most closely related to *V*. *stipulacea* in the section *Aconitifoliae*, and the two species were similar in that they had large stipules. Since *V*. *stipulacea* is a creeping plant cultivated as food, fodder, and green manure in Tamil Nadu, India [2], *V*. *khandalensis* might be used to improve *V*. *stipulacea* growth. *V*. *khandalensis* may also be useful as a genetic resource for other section *Ceratotoropis* crops, since the interspecific hybrid between this species and mung bean was obtained [47].

#### *Vigna marina* (Burm.) Merrill ssp. *oblonga* Padulosi

*V*. *marina* ssp. *oblonga* was proposed for the plants growing on the coastal zones of West Africa [19]. The phylogenetic tree using rDNA-ITS in this study confirmed that *V*. *marina* ssp. *oblonga* was more closely related to *V*. *luteola* than to *V*. *marina* ssp. *marina* (Fig 3), which was suggested by isozyme and RAPD analyses [20]. Additionally, phylogenetic trees suggest that there is a large intraspecific variation in *V*. *luteola*.

To address the evolution of *V*. *luteola* and *V*. *marina*, we need to consider *V*. *oblongifolia* A. Rich., a species closely related to these, although it was not included in this study. In *V*. *oblongifolia*, two botanical varieties have been described [25]. Phylogenetic trees in the previous studies have shown that *V*. *oblongifolia* var. *parviflora* is more closely related to *V*. *luteola* than to *V*. *marina*, and *V*. *oblongifolia* var. *oblongifolia* is more distant from these [48, 49]. This suggests that *V*. *marina* ssp. *oblonga* may be more closely related to *V*. *oblongifolia* var. *parviflora* than to *V*. *marina* ssp. *marina*. Therefore, the taxonomic treatment of *V*. *marina* ssp. *oblonga*, and *V*. *oblongifolia* var. *parviflora* should be reconsidered based on intra and inter-specific variations in *V*. *marina*, *V*. *luteola*, and *V*. *oblongifolia*.

Since there are no interspecific crossing barriers among *V*. *marina* ssp. *marina*, *V*. *marina* ssp. *oblonga*, and *V*. *luteola* [17, 50], and interspecific hybrid plants between *V*. *oblongifolia* and *V*. *luteola* were obtained [51], these are thought to form a primary gene pool. Therefore, to introduce the salinity and alkaline tolerance of *V*. *marina* into bambara groundnut, interspecific cross-compatibility should be investigated, taking into consideration the use of bridging species in the section *Vigna*. In Maxted et al. [16], there are 18 species listed in the section *Vigna*.

#### *Vigna vexillata* (L.) A. Rich

The wild forms of this species are widely distributed in pan-tropical regions, including Africa, Asia, Oceania, and America, and its swollen roots have been collected as food [52–54]. This species includes two domesticated forms that are morphologically distinct from each other. One is a twining plant without any taxonomic rank at an intraspecific level, which is cultivated in Bali, Indonesia [13]. Another is an erect plant named *V*. *vexillata* var. *macrosperma*, which is collected in Africa, Central America, and Australia. For both, the domestication origins are unknown.

In this study, the Indonesian domesticated form (accession ID-53) was found to be genetically differentiated from other species. This suggests that the Indonesian domesticated form, and *V*. *vexillata* var. *macrosperma* (accession ID-59), have been domesticated independently from different wild forms. This notion was also supported by the fact that a hybrid among the two domesticated forms was not obtained [55]. Moreover, there is an intraspecific crossing barrier between the Indonesian domesticated form and some wild forms [55]. Therefore, the ancestor of the Indonesian domesticated form is unknown.

Similarly, *V*. *vexillata* var. *lobatifolia* was found to be genetically differentiated from other species. This taxon was described originally as *V*. *lobatifolia* Baker [56], then classified as an independent species in the section *Pseudoliebrechtsia* [25], or the section *Plectrotropis* [5] in the subgenus *Plectrotropis*, and then given the current rank as botanical variety of *V*. *vexillata* based on isozyme polymorphisms [15, 16, 57]. However, since *lobatifolia* has a unique habitat (Namib Desert), and is morphologically distinct, we do not reject the taxonomic systems of Verdcourt [25] and Maréchal et al. [5], in which it was treated as an independent species. However, only nine accessions in five varieties of *V*. *vexillata* were analyzed for the subgenus *Plectrotropis* in this study, and thus further studies are required to systematize the taxonomy of this subgenus, and clarify the rank of the Indonesian domesticated forms and *V*. *vexillata* var. *lobatifolia*.

The natural habitat of *V*. *vexillata* was very diverse, including arid lands, coastal areas, acidic soil, and alkaline soil [16, 58, 59]. Some accessions have been reported to harbor flood resistance and pest resistance [60–63]. It is therefore believed that this species contains highly useful genetic resources to breed crops for agriculturally unfavorable lands.

### Future perspectives

In recent years, research on the use of wild relatives has been actively pursued. In addition to interspecific cross-breeding, new concepts have been proposed such as ‘Reverse Breeding’ [64], which involves regaining the crop stress tolerance, which has been lost in the breeding or domestication process, by backcrossing with the wild form. Another strategy is ‘Neo-Domestication’ [18], or the domestication of the stress-tolerant wild species that cannot be crossed with crop species. This process could be achieved by using mutation breeding, and mutant screening could be accelerated by TILLING, a screening method using the sequence information of domesticated genes. To advance these wild species breeding strategies, more information concerning the correct taxonomic placement, and genetic relationships among species, should be acquired to predict interspecific cross-compatibility, and to select an appropriate breeding strategy.
